# Efficacy and safety of mavacamten in treatment of hypertrophic cardiomyopathy: a systematic review and meta-analysis

**DOI:** 10.2144/fsoa-2023-0059

**Published:** 2023-09-19

**Authors:** Areeba Memon, Muhammad Omar Larik, Zoha Khan, Maryam Urooj, Areeka Irfan, Beena Kumari, Mehak Faisal, Rida Siddiqui, Moniba Tehrim, Ishaque Hameed

**Affiliations:** 1 Dow Medical College, Dow University of Health Sciences, Karachi, Pakistan; 2 Dow International Medical College, Dow University of Health Sciences, Karachi, Pakistan; 3 Karachi Medical & Dental College, Karachi, Pakistan

**Keywords:** cardiac myosin inhibitor, hypertrophic cardiomyopathy, mavacamten, MYK-461, nonobstructive cardiomyopathy, obstructive cardiomyopathy

## Abstract

**Aim:**

This meta-analysis was performed to assess the efficacy and safety of mavacamten in patients with hypertrophic cardiomyopathy.

**Methods & materials:**

A search was conducted using PubMed, Cochrane, and Scopus up to August 2022 for randomized studies reporting our pre-specified outcomes.

**Results:**

It was observed that mavacamten significantly improved New York Heart Association class (p < 0.009), Clinical Summary Score of the Kansas City Cardiomyopathy Questionnaire (p = 0.02), post-exercise left ventricular outflow tract gradient (p < 0.00001), functional end point (p = 0.05), and lowered septal reduction therapy rates (p < 0.00001). However, there were no significant differences in the ≥1 severe adverse events, ≥1 treatment-emergent adverse events, left ventricular volume index, left ventricular filling pressure, left ventricular end-diastolic volume index, and peak oxygen uptake (pVO_2_).

**Conclusion:**

Future large-scale trials are required to confirm our results and determine the long-term benefits and risks of mavacamten use in these patients.

## Plain language summary

Mavacamten is a recently introduced medication that relaxes the heart muscle and is indicated for patients with hypertrophic cardiomyopathy (a disease in which parts of the heart become thick and stiff). To determine the effectiveness and safety of this drug, the results of clinical trials were combined in order to produce an overall estimate. Overall, it was observed that mavacamten improved most functional parameters related to the heart and demonstrated no significant increases in the number of side effects. This suggests the effectiveness and safety of mavacamten, although further trials are needed to confirm our results.

## Tweetable abstract

The efficacy and safety of mavacamten, a novel drug, was assessed in patients of hypertrophic cardiomyopathy. It was noted that mavacamten significantly improved NYHA class, KCCQ-CSS score, post-exercise LVOT gradient, functional end point, and lowered SRT rates.

## Graphical abstract

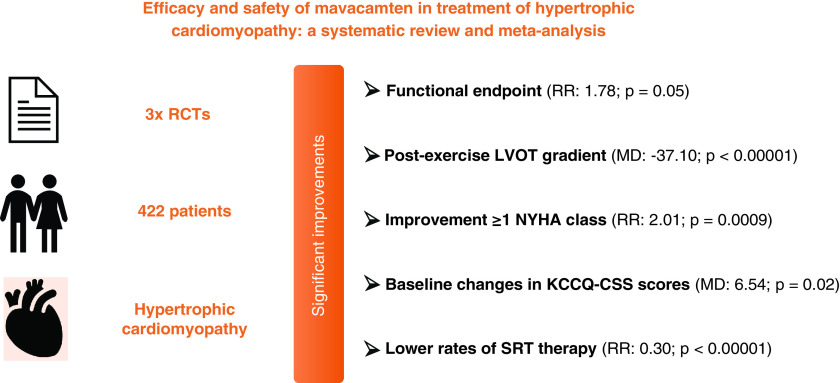

Hypertrophic cardiomyopathy (HCM) is a hereditary condition caused by a sarcomere protein gene mutation [1], affecting up to 0.2% of the population. It is characterized by left ventricular hypertrophy, fibrosis, hyper contractility, and reduced left ventricular volume [2]. Clinical presentation of hypertrophic cardiomyopathy may be asymptomatic or symptomatic ranging from angina, dyspnea, dizziness and palpitations [3]. However, exceptions do exist, as several patients may experience significant outcomes such as sudden cardiac death in young adults [4].

Current treatment guidelines indicate that the use of anti-arrhythmic drugs and β-blockers, alongside the use of anticoagulants, helps improve symptoms of thromboembolism in patients with HCM [5]. Although these treatment guidelines offer temporary symptomatic relief to patients, they fail to target the underlying pathophysiologic mechanisms manifesting in HCM, indicating the indispensability of a long-term treatment solution. In severe cases where patients are unresponsive to medical therapy – invasive procedures, such as septal ablation, may be necessary. However, the invasive nature of this procedure adds to morbidity and mortality. Additionally, procedural failure, repeated interventions, longer hospital stays, and a need for rather experienced hospitals to perform such procedures make it an undesirable option [6].

Mavacamten (MYK-461) is a novel drug placed in the class of cardiac myosin inhibitors – the first and only drug of its class to be approved by the US FDA in 2022 – as a form of targeted therapy for hypertrophic cardiomyopathy [7]. It is an allosteric inhibitor of cardiac-specific myosin adenosine triphosphatase. It helps in lessening hypercontractility by reducing excessive myosin-actin cross-bridging, which is believed to be an important contributor to HCM [7,8]. The primary mechanism of action of mavacamten is to reduce the steady-state ATPase activity by inhibiting the phosphate-release step in the “chemo-mechanical cycle”. This reduction results in a low release rate of bovine cardiac myosin-S1, which leads to a decrease in myocardial wall stress [9].

With HCM becoming increasingly refractory to the current drugs and treatment guidelines, patients are left with no choice but to pursue inexpedient surgical procedures (i.e., septal ablation) for relief [1]. Therefore, it is important to offer efficacious alternatives, such as mavacamten, to patients suffering from this chronic disease, as illustrated by recent studies [9–11]. However, the current number of limited randomized controlled trials (RCTs) conducted on mavacamten exhibit smaller sample sizes with marked heterogeneity. Moreover, given the varying degrees of severity and underlying pathophysiology between obstructive and non-obstructive hypertrophic cardiomyopathy, there has been a growing concern regarding the potential differences in the safety and efficacy of mavacamten across these two distinct patient populations. To address this issue, enhance the existing literature, and provide a holistic picture, we conducted a pre-specified subgroup meta-analysis to compare the effectiveness of mavacamten in individuals with obstructive versus non-obstructive hypertrophic cardiomyopathy.

## Methodology

### Data sources & search strategy

This meta-analysis was performed per the preferred reporting items for systematic review and meta-analyses (PRISMA) guidelines [12]. PubMed, Cochrane Library, and Scopus were searched from inception to August 2022. The detailed search strategy for each database is given in Supplementary Table 1. The reference lists of the retrieved articles were also reviewed for additional articles that might be relevant. The study has been reported in line with Assessing the methodological quality of systemic reviews (AMSTAR) guidelines [13].

### Study selection & eligibility criteria

All articles retrieved from the systematic search were exported to the EndNote reference library, version X8.1 (Clarivate Analytics), wherein duplicates were removed. Two independent investigators (AM and MU) assessed all articles at the title and abstract level, after which full text was read to confirm relevance. A third investigator (MF) was called to resolve any disagreements. We included articles only if the following pre-specified eligibility criteria were met: published randomized controlled trials; studies that compared outcomes after receiving mavacamten versus placebo; patients that were aged ≥18 years with a diagnosis of HCM; studies that reported at least one of the outcomes of interest. We excluded studies if they were case series, observational studies or non-English articles.

### Data extraction & quality of assessment

The following data were extracted from the included studies: patient baseline characteristics, improvement in ≥1 New York Heart Association (NYHA) class, change in peak oxygen uptake (pVO_2_), change in left ventricular outflow tract (LVOT) gradients, the Clinical Summary Score of the Kansas City Cardiomyopathy Questionnaire (KCCQ-CSS), (f) ≥1 total emergent adverse event (≥1 TEAE) such as chest pain, dizziness, non-sustained ventricular tachycardia, etc., and (g) ≥1 serious adverse event (≥1 SAE) such as atrial fibrillation, renal failure, systolic dysfunction, etc. were recorded. Two reviewers (ZK and AI) assessed the quality of the RCTs using the Cochrane Risk of Bias Tool for randomized controlled trials [14]. Any discrepancies were resolved by a third investigator (MOL) via discussion.

### Statistical analysis

Review Manager (RevMan version 5.3; Copenhagen: The Nordic Cochrane Centre, The Cochrane Collaboration, 2014) was used for all the statistical analyses with the results being pooled using a random effects model. A p-value was considered significant when it was ≤0.05. Continuous outcomes were assessed by mean difference while the risk ratios were employed to express the dichotomous outcome data, with 95% confidence intervals. To conduct heterogeneity evaluation, the Higgins I^2^ statistic value was used, and it was considered as significant when it was >50%. Moreover, we conducted the sensitivity analysis by employing the leave-one-out analysis to identify the trial causing significant heterogeneity. Additionally, we performed the subgroup analyses based on obstructive versus non-obstructive HCM.

## Results

### Literature search, characteristics & quality assessment

The initial search generated 421 potentially relevant articles up to August 2022. After removing duplicate studies and screening full-text articles, three relevant articles [9–11] were included in this systematic review and meta-analysis. The details of the screening process are summarized in the PRISMA flowchart in Supplementary Figure 1. A total of 422 patients were included in our meta-analysis, which consisted of 219 patients receiving the mavacamten treatment, and 203 patients receiving a placebo. Overall, the studies included in this meta-analysis were of high quality and illustrated a low risk of bias, as shown in Supplementary Table 2. The quality of the studies has been outlined in Supplementary Figure 2, with the summary highlighted in Supplementary Figure 3.

The baseline demographics of all included studies are illustrated in Table 1.

**Table 1.** Baseline characteristics of the included studies.

		EXPLORER-HCM [10]		VALOR-HCM [11]		MAVERICK-HCM [9]	
Study (year)		Olivotto I *et al.* (2020)		Desai MY *et al.* (2022)		Ho CY *et al.* (2020)	
		Mavacamten	Placebo	Mavacamten	Placebo	Mavacamten	Placebo
Sample size		123	128	56	56	40	19
Mavacamten (dose, mg)		Starting at 5 mg	–	5 mg, titrated up to 15 mg	–	5 mg, with 1 dose titration at week 6	–
Follow-up duration, weeks		30		16		16	
Mean age, years		58·5 (12.2)	58·5 (11.8)	59.8 (14.2)	60.9 (10.5)	54 (14.6)	53.8 (18.2)
Sex	Male	66	83	29	28	19	6
	Female	57	45	27	28	21	13
Duration of HCM, years		–	–	7.5 (9.4)	6.7 (7.4)	–	–
Medical History	Family history of HCM	33	36	17	15	–	–
	A fib	12	23	11	8	–	–
	Hypertension	57	53	36	34	–	–
	Syncope or Pre-syncope	–	–	29	30	–	–
	SRT	11	8	–	–	–	–
	Hyperlipidemia	27	39	–	–	–	–
	CAD	12	6	–	–	–	–
	Obesity	15	14	–	–	–	–
	Type 2 diabetes	6	7	–	–	–	–
	Asthma	17	11	–	–	–	–
	COPD	2	3	–	–	–	–
	ICD	27	29	9	10	–	–
NYHA Classification	Class II	88	95	4	4	33	13
	Class III or higher	35	33	52	52	7	6
Background HCM therapy	βB	94	95	45	39	25	12
	CaCB	25	17	16	23	10	3
Echocardiographic parameters LVOT gradient, mm Hg	LVOT gradient, Rest, mm Hg	52 (29)	51 (32)	51.2 (31.4)	46.3 (30.5)	–	–
	LVOT gradient, Valsalva, mm Hg	72 (32)	74 (32)	75.3 (30.8)	76.2 (29.9)	–	–
	LVOT gradient, Post-exercise, mm Hg	86 (34)	84 (36)	82.5 (34.7)	85.2 (37)	–	–
	LVEF, %	74 (6)	74 (6)	67.9 (3.7)	68.3 (3.2)	68.7 (5.5)	66.4 (7.7)
	Left atrial diameter, mm	42 (5)	42 (6)	–	–	–	–
	Maximum left ventricular wall thickness, mm	20 (4)	20 (3)	–	–	20.6 (4)	18.8 (3.5)
	Left atrial volume index, ml/m2	40 (12)	41 (14)	41.3 (16.5)	40.9 (15.2)	37.3 (13)	40.8 (15.2)
Pathogenic or likely pathogenic HCM gene variant/HCM genetic testing consented to or performed		28/90	22/100			14/28	8/12
Laboratory measurements (Drug/Control)	NT-proBNP, ng/l	777 (136)	616 (108)	724 (1201.5)	743 (682.2)	821 (811.5)	914 (876.2)
	Cardiac troponin I, ng/l	–	–	17.3 (18.2)	12.9 (14.7)	23 (408.2)	20 (117.9)
	Cardiac troponin T, μg/l	–	–	0.014 (0.007)	0.011 (0.008)	–	–
pVO2, ml/kg per min		18.9 (4.9)	19.9 (4.9)	–	–	20.4 (6)	17.9 (5.1)

A fib: Atrial fibrillation; βB: Beta blocker; CaCB: Calcium Channel Blocker; CAD: Coronary artery disease; COPD: Chronic obstructive pulmonary disease; HCM: Hypertrophic cardiomyopathy; ICD: Implantable cardioverter defibrillator; LVOT: Left ventricular outflow tract; LVEF: Left ventricular ejection fraction; NYHA: New York Heart Association; NT-proBNP: N-terminal pro B-type natriuretic peptide; pVO_2_: Peak venous oxygen consumption; SRT: Septal reduction therapy.

### Efficacy of mavacamten

**Figure 1.**
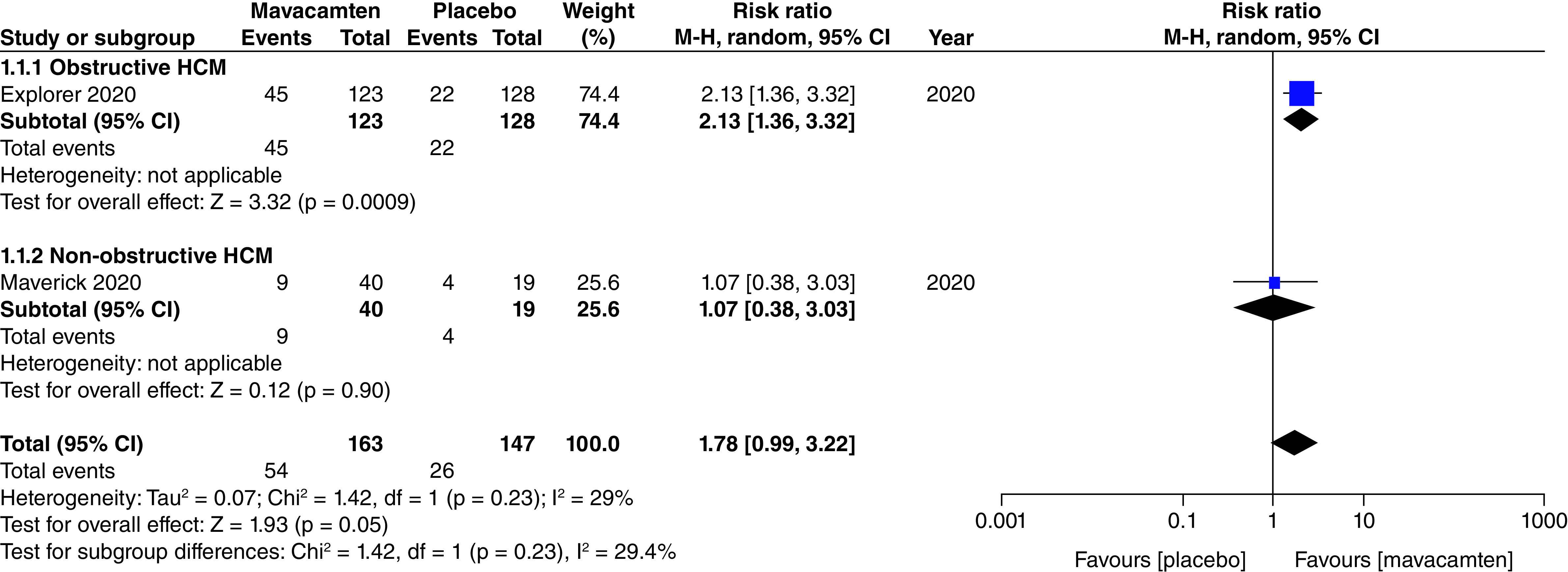
Forest plot of composite functional end point. CI: Confidence interval; HCM: Hypertrophic cardiomyopathy.

**Figure 2.**
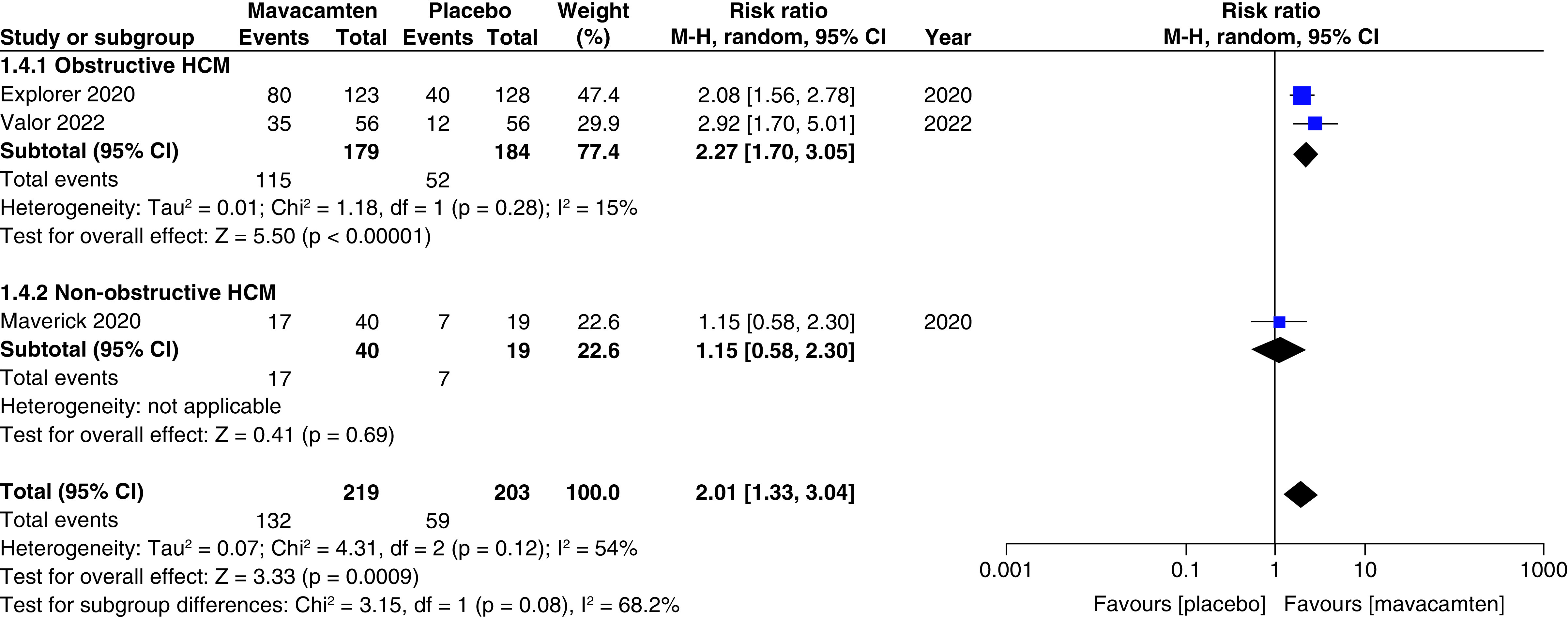
Forest plot of ≥1 New York Heart Association class. CI: Confidence interval; HCM: Hypertrophic cardiomyopathy.

**Figure 3.**
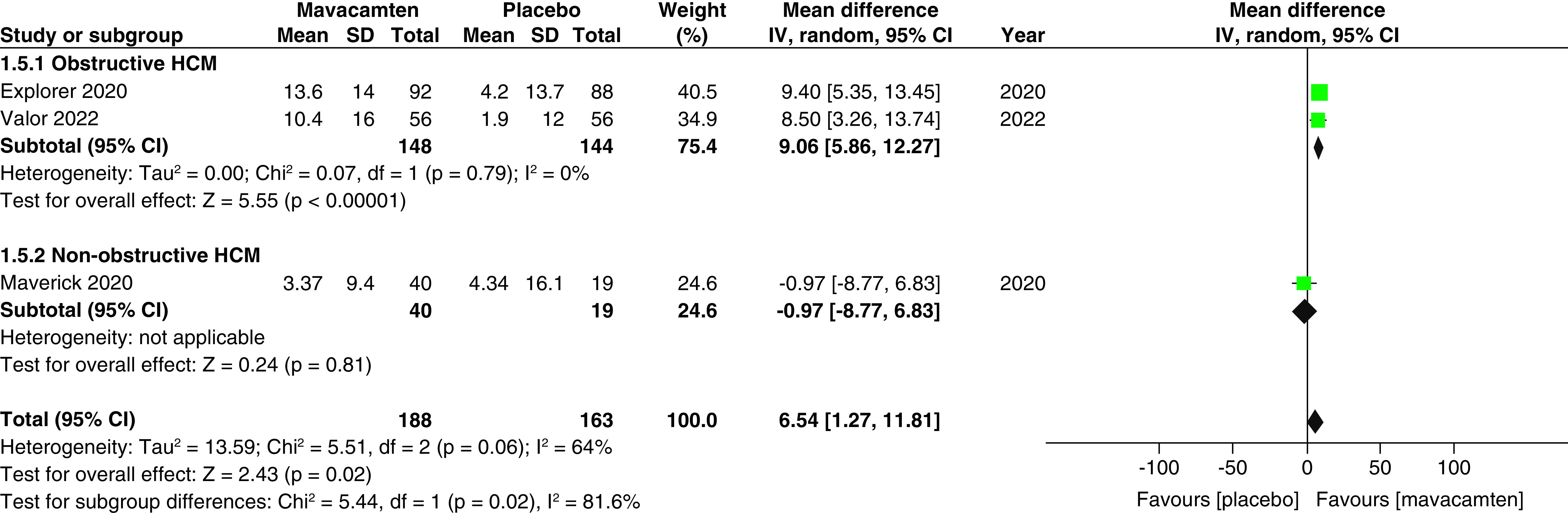
Forest plot of changes from baseline in Clinical Summary Score of the Kansas City Cardiomyopathy Questionnaire score. CI: Confidence interval; HCM: Hypertrophic cardiomyopathy; SD: Standard deviation.

**Figure 4.**
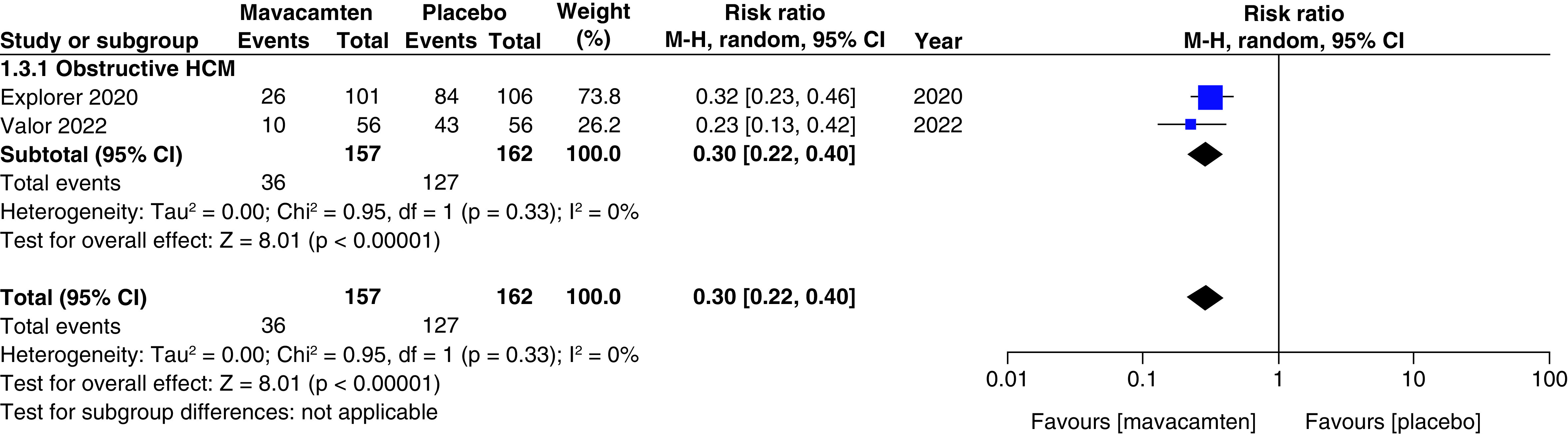
Forest plot of eligibility for septal reduction therapy. CI: Confidence interval; HCM: Hypertrophic cardiomyopathy.

**Figure 5.**
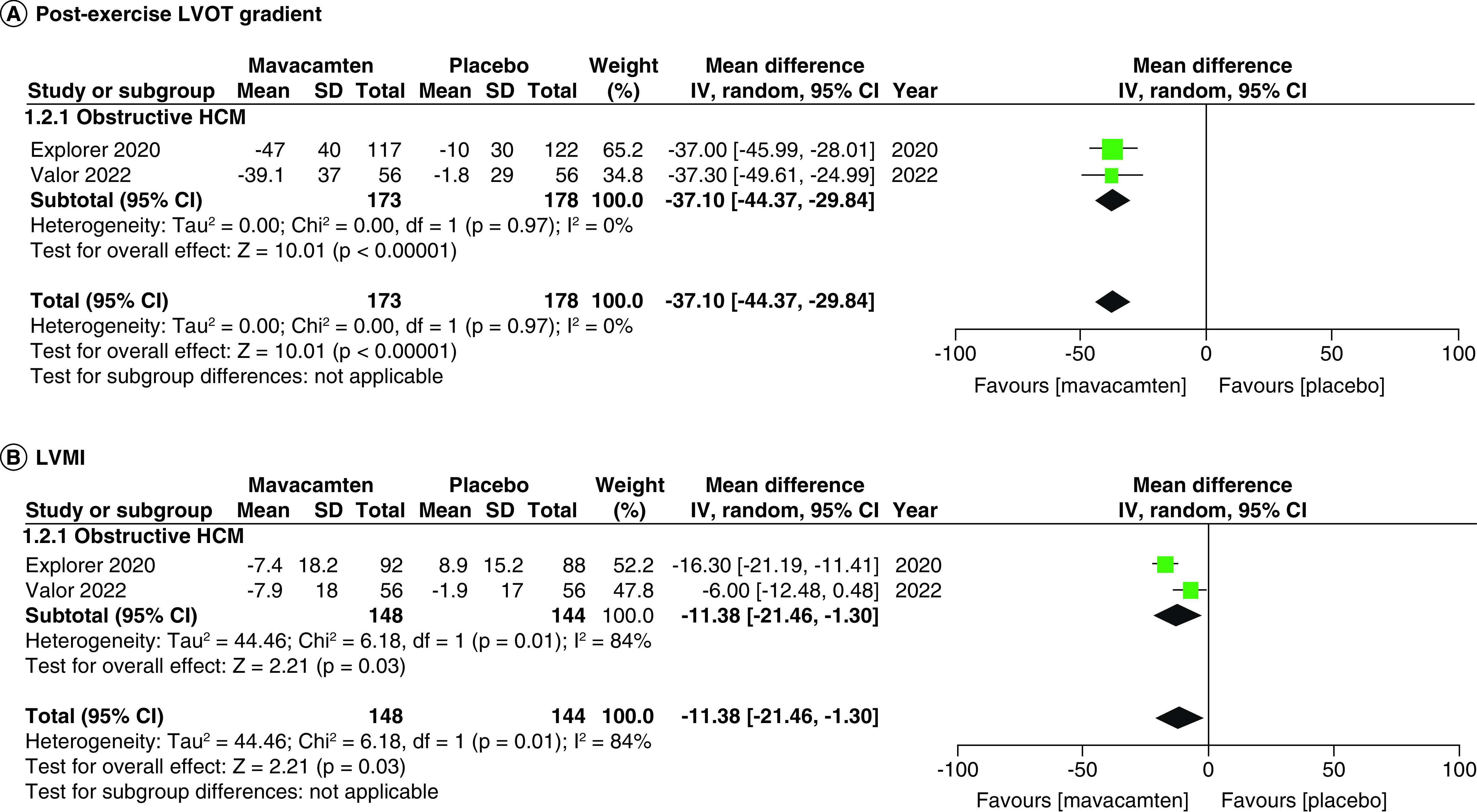
Forest plots of changes from baseline in post-exercise left ventricular outflow tract gradient and left ventricular mass index. **(A)** Refers to the changes from baseline in post-exercise left ventricular outflow tract gradient. **(B)** Refers to the changes from baseline in left ventricular mass index. CI: Confidence interval; HCM: Hypertrophic cardiomyopathy; SD: Standard deviation.

**Figure 6.**
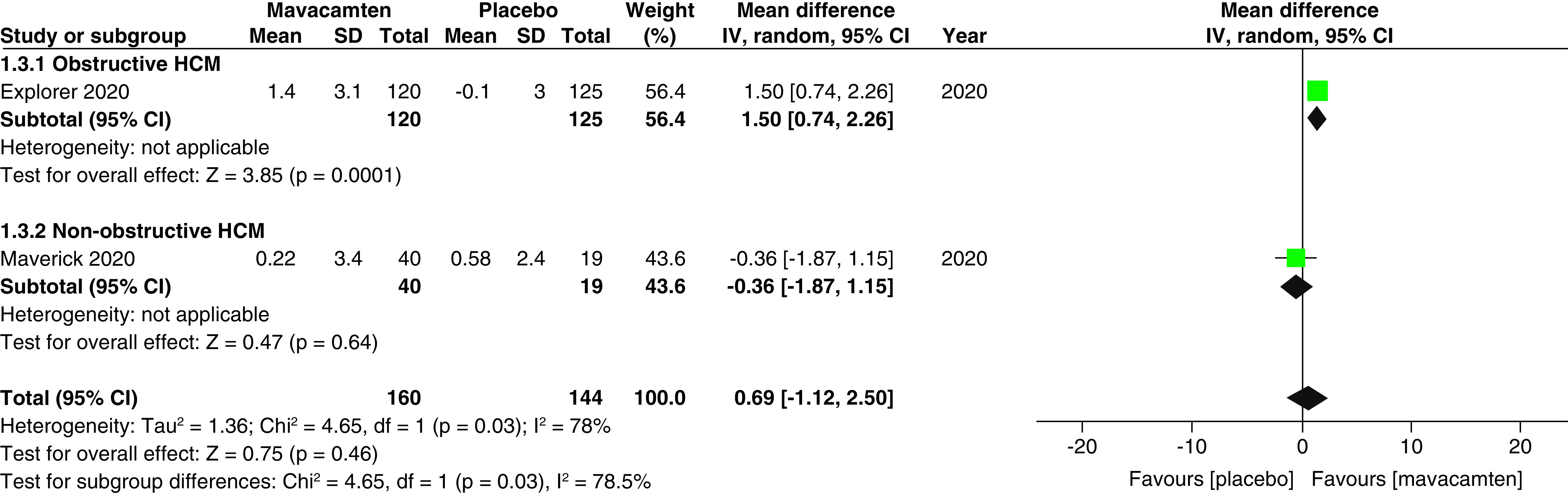
Forest plot of changes from baseline in peak oxygen uptake (pVO_2_). CI: Confidence interval; HCM: Hypertrophic cardiomyopathy; SD: Standard deviation.

**Figure 7.**
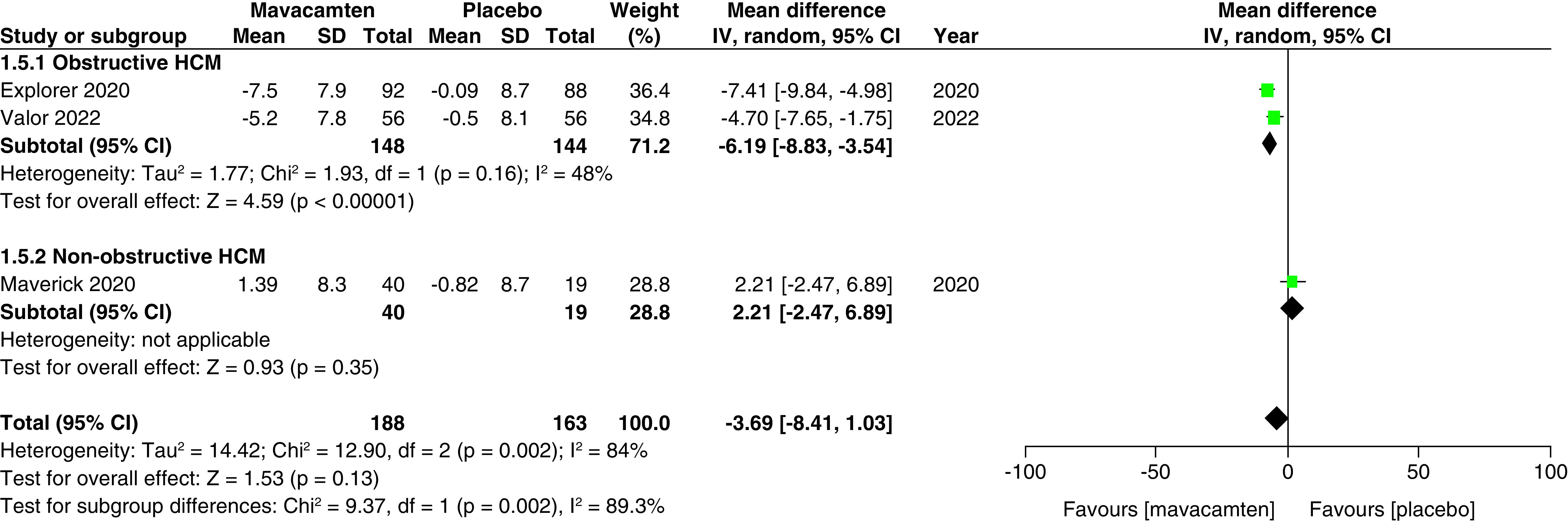
Forest plot of changes from baseline in left atrial volume index. CI: Confidence interval; HCM: Hypertrophic cardiomyopathy; SD: Standard deviation.

**Figure 8.**
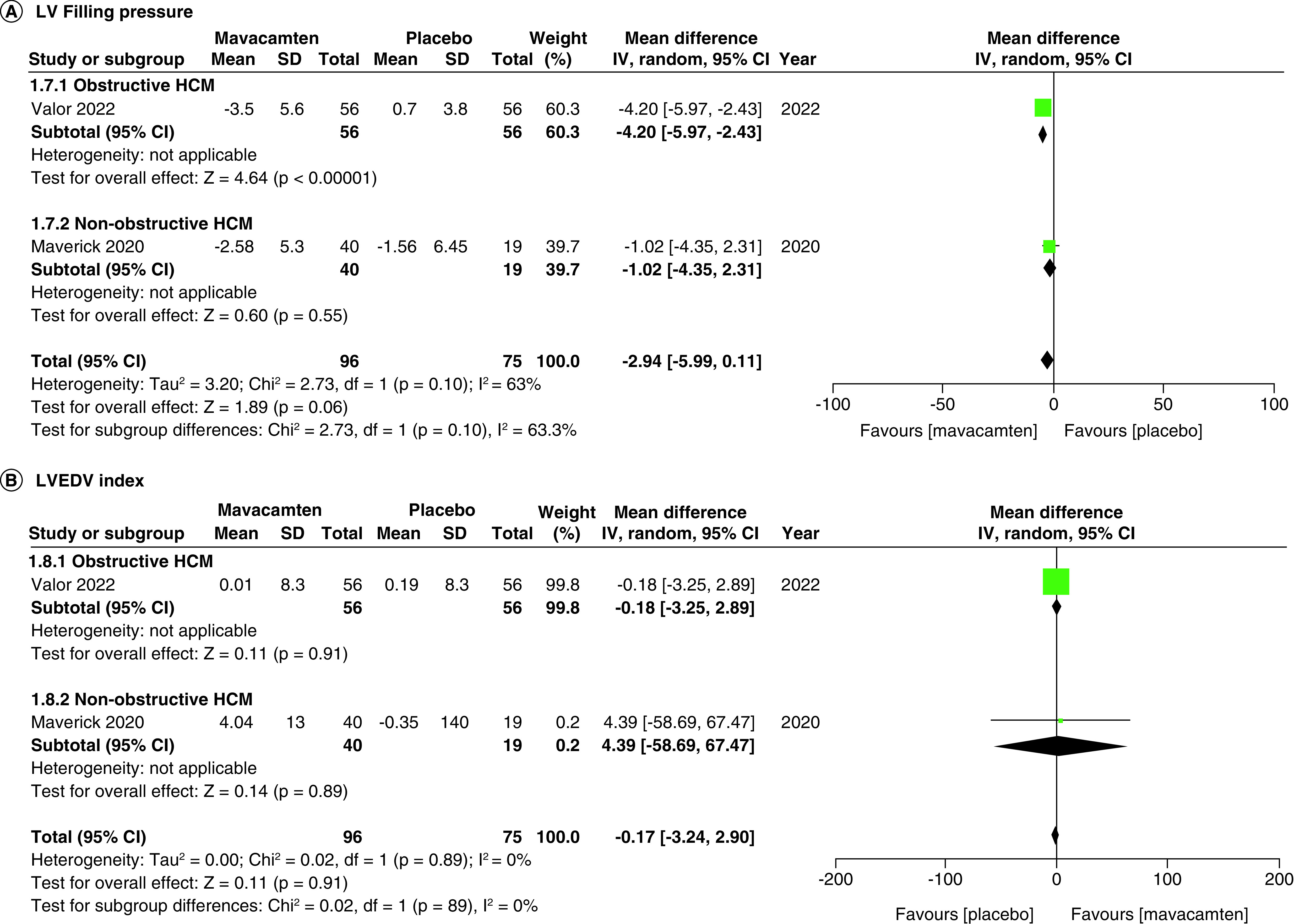
Forest plots of changes from baseline in left ventricular filling pressure and left ventricular end-diastolic volume index. **(A)** Refers to the changes from baseline in left ventricular filling pressure. **(B)** Refers to the changes from baseline in left ventricular end-diastolic volume index. CI: Confidence interval; HCM: Hypertrophic cardiomyopathy; SD: Standard deviation.

Two studies of our analysis reported composite functional end points. The aggregated results demonstrated a significant relationship with the number of participants meeting the composite functional end point when mavacamten was compared with placebo (RR: 1.78; 95% CI: 0.99, 3.22; p = 0.05; I^2^ = 0%; Figure 1). Pooling the data reporting improvement in ≥1 NYHA class revealed that mavacamtem was associated with significant improvement as compared with the placebo (RR: 2.01; 95% CI: 1.33, 3.04; p = 0.0009; I^2^ = 54%, Figure 2). The combined estimates for change from baseline in KCCQ-CSS score revealed a significant improvement with mavacamten (mean difference: 6.54, 95% CI: 1.27, 11.81; p = 0.02, Figure 3) with high heterogeneity of I^2^ = 64%. Additionally, patients had significantly lower rates of eligibility for septal reduction therapy (SRT) therapy when receiving mavacamten, as compared with placebo (RR: 0.30; 95% CI: 0.22, 0.40; p < 0.00001; Figure 4). Baseline changes of post-exercise LVOT gradient had revealed a significant improvement favoring mavacamten when compared with placebo (mean difference: -37.10; 95% CI: -44.37, -29.84; p < 0.00001; Figure 5A). However, baseline changes of Left Ventricular Mass Index (LVMI) did not demonstrate a significant difference when mavacamten was compared with placebo (mean difference: -11.38; 95% CI: -21.46, -1.30; p = 0.03; I^2^ = 83%; Figure 5B). With respect to the changes of pVO_2_ from baseline, a non-significant relationship was illustrated when mavacamten was compared with placebo (mean difference: 0.69; 95% CI: -1.12, 2.50; p = 0.46; I^2^ = 78%; Figure 6). With regards to change from baseline in Left Atrial Volume Index (LAVI), there was no statistically significant change seen between the two groups, (mean difference: -3.69, 95% CI: -8.41, 1.03; p = 0.13; I^2^ = 84%; Figure 7). Furthermore, when assessing for change from baseline in LV filling pressures, no statistically significant relationship was illustrated between the two groups (mean difference: -2.94; 95% CI: -5.99, 0.11; p = 0.06; I^2^ = 63%; Figure 8A). A similar effect was observed for the change from baseline in Left Ventricular End-Diastolic Volume (LVEDV) Index, where the difference failed to reach statistical significance (mean difference: -0.17; 95% CI: -3.24, 2.90; p = 0.91, I^2^ = 29%, Figure 8B).

### Safety of mavacamten

**Figure 9.**
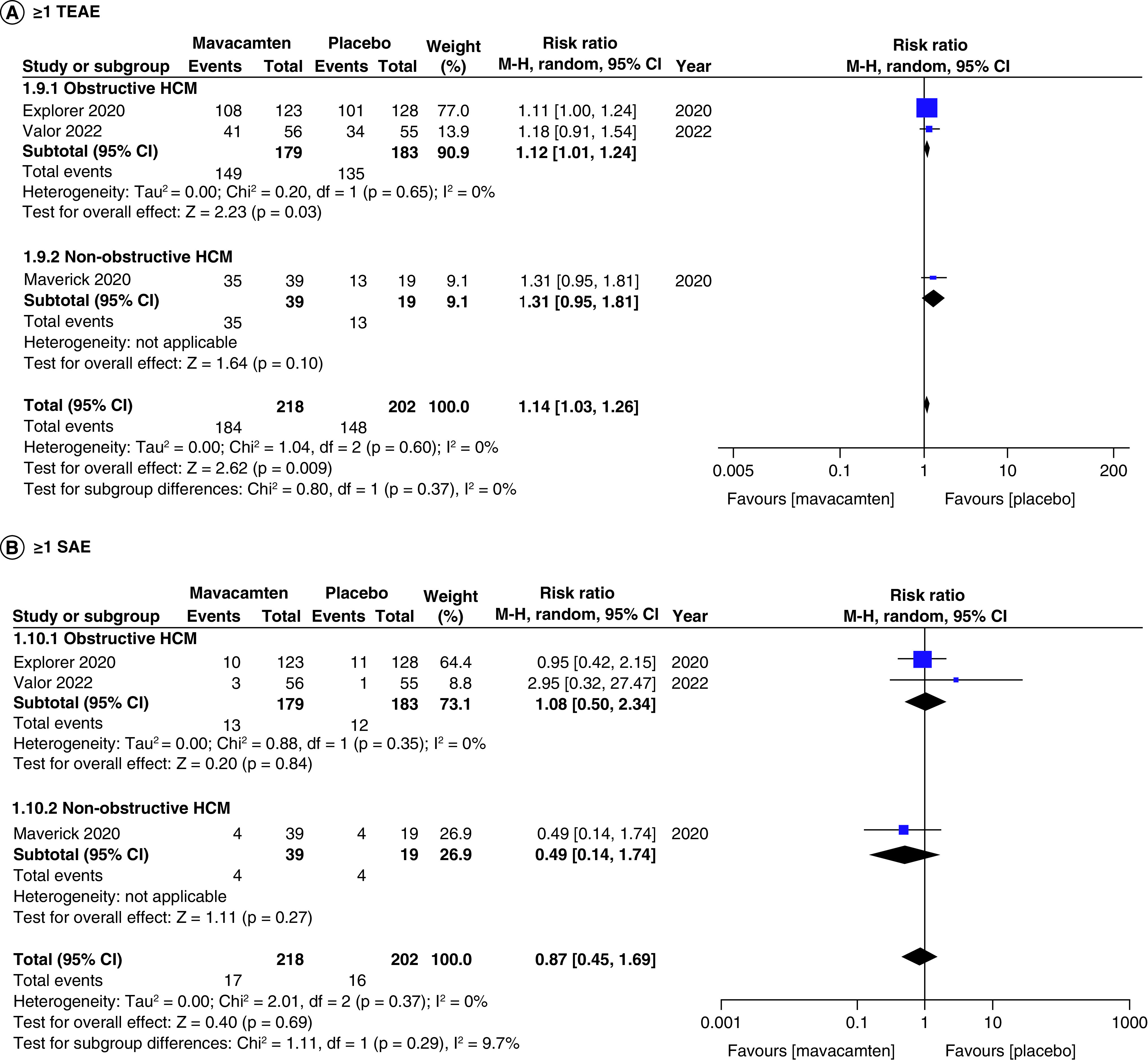
Forest plots of participants with ≥1 treatment-emergent adverse events and ≥1 severe adverse events. **(A)** Refers to incidence of ≥1 treatment-emergent adverse event. **(B)** Refers to incidence of ≥1 severe adverse event. CI: Confidence interval; HCM: Hypertrophic cardiomyopathy.

**Figure 10.**
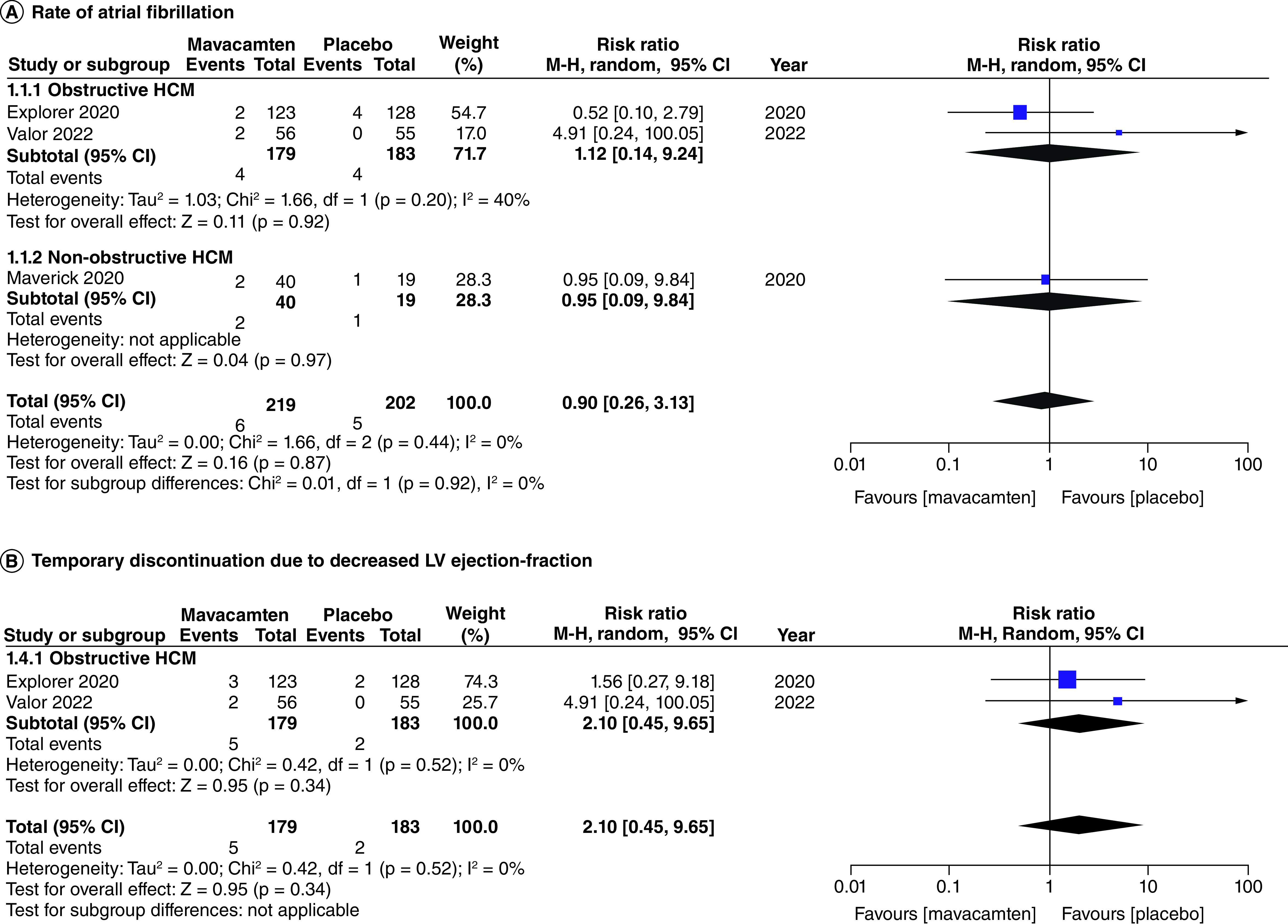
Forest plots of rate of atrial fibrillation and temporary discontinuation due to decreased left ventricular ejection-fraction. **(A)** Refers to the rate of atrial fibrillation. **(B)** Refers to the incidence of temporary discontinuation due to decreased left ventricular ejection-fraction. CI: Confidence interval; HCM: Hypertrophic cardiomyopathy.

All studies of our analysis reported the data of participants with ≥1 treatment-emergent adverse event (≥1 TEAE) and ≥1 severe adverse event (≥1 SAE). The aggregated results demonstrated that mavacamten was associated with higher rates of ≥1 TEAE (RR: 1.14; 95% CI: 1.03, 1.26; p = 0.009; Figure 9A). However, the pooled results for ≥1 SAE illustrated that the difference in incidence between mavacamten and placebo was not significant (RR: 0.87; 95% CI: 0.45, 1.69; p = 0.69; Figure 9B). One of the adverse events included atrial fibrillation, which did not display a significant difference when mavacamten was compared with placebo (RR: 0.90; 95% CI: 0.26, 3.13; p = 0.87; Figure 10A). Furthermore, there was no significant difference observed between mavacamten and placebo with respect to the temporary discontinuation of treatment due to LV ejection fraction falling below threshold (RR: 2.10; 95% CI: 0.45, 9.65; p = 0.34; Figure 10B).

### Subgroup analyses

Two cohorts (participants with obstructive HCM, and participants with non-obstructive HCM) were created for the subgroup analyses. There was a non-significant relationship observed between the subgroups for the number of participants meeting the composite functional end point, improvement in ≥1 NYHA classification, changes from baseline in LV filling pressure, changes from baseline in LVEDV index, patients with ≥1 TEAE, patients with ≥1 SAE, and patients with atrial fibrillation as an adverse event.

However, there was a significant difference observed between changes from the baseline in pVO_2_, with patients of obstructive HCM demonstrating a greater change than patients of non-obstructive HCM (mean difference: 1.50 vs -0.36; p = 0.03; Figure 6). There was a significant difference observed between KCCQ-CSS scores, with patients of obstructive HCM showing greater scores than patients of non-obstructive HCM (mean difference: 9.06 vs -0.97; p = 0.02; Figure 3). There was a significant difference observed between changes from baseline in LAVI, with non-obstructive patients showing greater changes when compared with patients of obstructive HCM (mean difference: 2.21 vs -6.19; p = 0.002; Figure 7).

## Discussion

The reasonable hesitancy with the use of mavacamten in clinical practice can be attributed to its recent premiere into the medical field, owing to skepticism regarding its efficacy and safety when compared with current standards of practice. With limited data procured by each individual RCT, this provided the perfect opportunity to conduct a systematic review and meta-analysis on the efficacy and safety of mavacamten in the treatment of HCM. Our study provides a comprehensive analysis by pooling data from three RCTs, which included a total of 422 patients. The analysis generated compelling evidence supporting the favorable efficacy of mavacamten toward end point outcomes such as a ≥1 NYHA class, KCCQ-CSS scores, post-exercise LVOT gradient, composite functional end point, and baseline changes in LVMI. In terms of safety, a slight increase in ≥1 treatment-emergent adverse events (TEAEs) were noted, while no significant differences were observed in ≥1 serious adverse events (SAEs), number of atrial fibrillation events, and in patients temporarily discontinuing treatment due to fall in LV ejection-fraction below threshold. To the best of our knowledge, this is the most comprehensive meta-analysis conducted to date, featuring several outcomes of interest. Additionally, a pre-defined subgroup analysis based on obstructive HCM versus non-obstructive HCM was performed, enabling the direct comparison between both cohorts, allowing researchers and clinicians to identify the suitability and appropriateness of mavacamten in certain patient populations.

Mavacamten is a cardiac sarcomere inhibitor that reduces excessive myocardial contractility which helps improve LV diastolic filling, reduce LVOT obstruction, and enhance ventricular lusitropy [11]. The resultant improved LV compliance leads to alleviation in symptom severity in HCM patients at rest and on exertion as is reflected by an improvement in ≥1 NYHA class and post-exercise LVOT gradient. Moreover, the favorable effect of mavacamten on a patient's functional limitations, quality of life as well as symptom burden manifested into a better KCCQ-CSS score. This meta-analysis distinguishes itself from previous studies by incorporating novel outcome measures, including post-exercise LVOT gradient, change in KCCQ-CSS scores, and LVMI [15,16]. The analysis highlights favorable changes in these parameters in the mavacamten group, indicating improved exercise tolerance, enhanced quality of life, and potential regression of hypertrophy. Furthermore, the study reveals that mavacamten treatment did not result in any changes requiring temporary drug discontinuation, emphasizing its tolerability and safety.

One significant finding is the efficacy of mavacamten in reducing eligibility for septal reduction therapy (SRT), an invasive procedure often recommended for severe HCM. This suggests that mavacamten can provide a favorable avenue for symptom relief in HCM patients, potentially avoiding the need for expensive and invasive procedures associated with several complications. The mechanism of mavacamten can result in LV systolic dysfunction [17], which may explain the slight yet significant increase in the occurrence of ≥1 TEAE noted in our study. Thus, close surveillance and timely, appropriate management are imperative. Despite this, the drug was generally well-tolerated by most patients, with no severe adverse effects warranting permanent drug discontinuation in VALOR-HCM [11]. However, temporary discontinuation from the ongoing study was observed, due to decreased LV ejection-fraction below the threshold, although, our analysis revealed that mavacamten was not subject to increased rates of temporary discontinuation, adding to its safety profile. Common TEAEs included dizziness/fatigue, palpitations, and dyspnea, with the most common SAE being atrial fibrillation, reported by all participating studies [9–11]. Despite atrial fibrillation being the most frequent SAE, the mavacamten cohort did not experience significantly greater incidence of atrial fibrillation when compared with placebo. This exemplifies the safety of mavacamten, which indicates that there were no SAEs of concern with regards to the patients receiving the active drug. Additionally, while there was a negligible difference in the total number of TEAEs based on dosage, with 88.9% in the 200 ng/ml cohort versus 90.5% in the 500 ng/ml cohort, however, a pronounced occurrence of atrial fibrillation in the 500 ng/ml cohort instigates a source of concern, with three cases reported compared with zero cases reported in the 200 ng/ml cohort [9]. Therefore, further studies are strongly encouraged to assess the relationship between the dosage of mavacamten and its safety and tolerability profile.

While our study did not demonstrate a significant increase in the risk of ≥1 SAE, long-term clinical trials such as A Long-Term Safety Extension Study of Mavacamten in Adults Who Have Completed MAVERICK-HCM or EXPLORER-HCM (NCT03723655) [18] and Extension Study of Mavacamten (MYK-461) in Adults with Symptomatic Obstructive Hypertrophic Cardiomyopathy Previously Enrolled in PIONEER (PIONEER OLE) (NCT03496168) [19] are currently underway. These studies aim to provide enhanced clarity regarding the magnitude of adverse drug effects in the long term, due to extended follow-up durations offered compared with the relatively short follow-up durations of current trials such as MAVERICK-HCM and VALOR-HCM.

There was substantial heterogeneity observed within ≥1 NYHA class improvement, KCCQ-CSS score, and baseline changes in LAVI. This can be attributed to the study conducted by Ho CY *et al.* (MAVERICK-HCM), as the subgroup analysis revealed significant improvement when studies were categorized on basis of obstructive HCM versus non-obstructive HCM populations [9]. Moreover, this divergence may also be due to exclusion of patients with an LVOT gradient greater than 30 mm Hg. Additionally, MAVERICK-HCM further stratified the drug group into two subgroups, each receiving different doses of mavacamten. Additionally, variations in disease duration and dose-titration of the drug, concomitant medications, adherence, follow-up duration, and center-specific practices could contribute to the disparity. It is also important to acknowledge that there was a baseline imbalance in the prescription of beta-blockers and calcium channel blockers, which have inotropic and chronotropic effects, possibly acting as a confounder in the study, and may contribute to heterogeneity within our results. Further research and adjustments for these potential confounding factors are warranted to strengthen the reliability and generalizability of the results.

### Future directions

With the recent introduction and approval of mavacamten as a novel drug for treating HCM patients, it is important to sustain the momentum of research being conducted to arrive at a valid conclusion, and whether mavacamten will be able to truly replace the current invasive techniques, such as SRT. Moreover, the complex pathophysiology of HCM results in reasonable hesitance and therefore warrants further comprehensive research, to unfold certain unaddressed questions [20]. First, the current RCTs do not evaluate the efficacy of mavacamten in reducing HCM-related clinical outcomes, such as atrial fibrillation or heart failure, indicating the necessity and the value of ongoing extension studies assessing mavacamten. Second, with the MAVERICK-HCM trial being the only RCT reporting placebo-controlled results on the non-obstructive HCM population; researchers are greatly encouraged to assess the differences in efficacy in such patients, especially as certain outcomes within this meta-analysis observed significant differences between both cohorts. Third, while adults seem to demonstrate favorable results, it is important to consider whether mavacamten will provide similar benefits to children to limit disease progression, and simultaneously minimize the risk of poor prognostic outcomes in the future. Finally, the possible utilization of mavacamten as a prophylactic agent for those predisposed to the development of HCM must be evaluated, in order to limit disease progression, diminish the risk of poor outcomes, and improve the future quality of life of patients with relevant genotypes. These research questions are essential in depicting the complete landscape of mavacamten's possible use for HCM patients, in the bid to evade the existing guidelines of invasive procedures.

### Limitations of study

While our meta-analysis yielded valuable insights into mavacamten's efficacy and safety, we acknowledge the limitation of a small overall sample size with a limited follow-up duration due to the inclusion of only three studies. Additionally, not all studies reported on the parameters being assessed, which further restricted our sample size for certain outcomes. Furthermore, due to the lack of stratification based on background medical or surgical therapies, it was not possible to assess their impact on our results. Moreover, the innate differences in the physiology of obstructive HCM and non-obstructive HCM may affect the generalizability of our findings, highlighting the need for further trials to effectively compare the two types. Another limitation of our paper was that only two included studies reported data on the LVMI, pVO2 and LV filling pressures, therefore, we were not able to perform a subgroup/sensitivity analysis to find the cause of heterogeneity. Despite this constraint, our findings still provide preliminary evidence supporting mavacamten's potential in treating hypertrophic cardiomyopathy.

## Conclusion

In conclusion, this meta-analysis of 422 patients showed that mavacamten has the potential to significantly impact outcomes in HCM patients, particularly improvement in ≥1 NYHA class, KCCQ-CSS scores, composite functional end point, post-exercise LVOT gradients, LVMI changes, and lowered SRT rate, with its own set of ≥1 TEAE, setting the stage for future trials to confirm our findings and test this drug in more HCM patients, averting the route from the conventional sub-optimal or invasive treatment options to a non-invasive and optimal treatment option.

**Summary points**

- Mavacamten, a novel cardiac myosin inhibitor, for hypertrophic cardiomyopathy.
- Shows improvement in >1 New York Heart Association (NYHA) class, Clinical Summary Score of the Kansas City Cardiomyopathy Questionnaire, and post-exercise left ventricular outflow tract gradient, among other included outcomes.
- Slight increase in the risk of >1 treatment-emergent adverse events.
- Large-scale trials needed to evaluate long-term risks versus benefits.
- Is there hope for change from invasive septal reduction therapy to non-invasive medical treatment?
