# Supplementary material for: Efficacy and safety of mavacamten in treatment of hypertrophic cardiomyopathy: a systematic review and meta-analysis
Source: Future Sci OA. 2023 Sep 19;9(10):FSO898. doi: 10.2144/fsoa-2023-0059 (PMC10518811; doi:10.2144/fsoa-2023-0059)
Supplement: Supplementary file 1 [file fsoa-09-898-s1.pdf]

Supplemental Table 1. Search Strategy

SEARCH STRATEGY	DATABASE	CITATIONS
("myk 461"[Supplementary Concept] OR "myk 461"[All Fields] OR "mavacamten"[All Fields] OR ("myk 461"[Supplementary Concept] OR "myk 461"[All Fields] OR "myk 461"[All Fields]) OR (("myosin s"[All Fields] OR "myosine"[All Fields] OR "myosins"[MeSH Terms] OR "myosins"[All Fields] OR "myosin"[All Fields]) AND ("inhibit"[All Fields] OR "inhibitable"[All Fields] OR "inhibite"[All Fields] OR "inhibited"[All Fields] OR "inhibites"[All Fields] OR "inhibiting"[All Fields] OR "inhibition, psychological"[MeSH Terms] OR ("inhibition"[All Fields] AND "psychological"[All Fields]) OR "psychological inhibition"[All Fields] OR "inhibition"[All Fields] OR "inhibitions"[All Fields] OR "inhibitive"[All Fields] OR "inhibits"[All Fields])) OR (("myosin s"[All Fields] OR "myosine"[All Fields] OR "myosins"[MeSH Terms] OR "myosins"[All Fields] OR "myosin"[All Fields]) AND ("antagonists and inhibitors"[MeSH Subheading] OR ("antagonists"[All Fields] AND "inhibitors"[All Fields]) OR "antagonists and inhibitors"[All Fields] OR "inhibitors"[All Fields] OR "inhibitor"[All Fields] OR "inhibitor s"[All Fields])) OR ("Actin-myosin"[All Fields] AND ("antagonists and inhibitors"[MeSH Subheading] OR ("antagonists"[All Fields] AND "inhibitors"[All Fields]) OR "antagonists and inhibitors"[All Fields] OR "inhibitors"[All Fields] OR "inhibitor"[All Fields] OR "inhibitor s"[All Fields]))) AND ("cardiomyopathy, hypertrophic"[MeSH Terms] OR ("cardiomyopathy"[All Fields] AND "hypertrophic"[All Fields]) OR "hypertrophic cardiomyopathy"[All Fields] OR ("hypertrophic"[All Fields] AND "cardiomyopathy"[All Fields]) OR ("health cost manage"[Journal] OR "health care manag frederick"[Journal] OR "hcm"[All Fields]) OR ("cardiomyopathy, hypertrophic"[MeSH Terms] OR ("cardiomyopathy"[All Fields] AND "hypertrophic"[All Fields]) OR "hypertrophic cardiomyopathy"[All Fields] OR ("obstructive"[All Fields] AND "hypertrophic"[All Fields] AND "cardiomyopathy"[All Fields]) OR "obstructive hypertrophic cardiomyopathy"[All Fields]) OR ("cardiomyopathy, hypertrophic"[MeSH Terms] OR ("cardiomyopathy"[All Fields] AND "hypertrophic"[All Fields]) OR "hypertrophic cardiomyopathy"[All Fields] OR "hocm"[All Fields]) OR ("Non-obstructive"[All Fields] AND ("cardiomyopathy, hypertrophic"[MeSH Terms] OR ("cardiomyopathy"[All Fields] AND "hypertrophic"[All Fields]) OR "hypertrophic cardiomyopathy"[All Fields] OR ("hypertrophic"[All Fields] AND "cardiomyopathy"[All Fields]))) OR (("nonobstructed"[All Fields] OR "nonobstructing"[All Fields] OR "nonobstruction"[All Fields] OR "nonobstructive"[All Fields]) AND ("cardiomyopathy, hypertrophic"[MeSH Terms] OR ("cardiomyopathy"[All Fields] AND "hypertrophic"[All Fields]) OR "hypertrophic cardiomyopathy"[All Fields] OR ("hypertrophic"[All Fields] AND "cardiomyopathy"[All Fields]))))	PUBMED	236
(Mavacamten OR MYK-461 OR Myosin inhibition OR Myosin inhibitor OR Actin-myosin inhibitor) AND (Hypertrophic cardiomyopathy OR HCM OR Obstructive hypertrophic cardiomyopathy OR HOCM OR Non-obstructive hypertrophic cardiomyopathy OR Nonobstructive hypertrophic cardiomyopathy)	COCHRANE CENTRAL	34
TITLE-ABS-KEY (("Mavacamten" OR "MYK-461" OR "Myosin inhibition" OR "Myosin inhibitor" OR "Actin-myosin inhibitor") AND ("Hypertrophic cardiomyopathy" OR "HCM" OR "Obstructive hypertrophic cardiomyopathy" OR "HOCM" OR "Non-obstructive hypertrophic cardiomyopathy" OR "Nonobstructive hypertrophic cardiomyopathy"))	SCOPUS	151
