# Supplementary material for: Efficacy and safety of mavacamten in treatment of hypertrophic cardiomyopathy: a systematic review and meta-analysis
Source: Future Sci OA. 2023 Sep 19;9(10):FSO898. doi: 10.2144/fsoa-2023-0059 (PMC10518811; doi:10.2144/fsoa-2023-0059)
Supplement: Supplementary file 2 [file fsoa-09-898-s2.pdf]

Supplemental Table 2. Cochrane Risk of Bias Assessment

	**Cochrane Risk-of-Bias Tool**		
	Bias	Risk of bias	Author judgement
**EXPLORER-HCM TRIAL**	Random sequence generation (selection bias)	Low Risk	Patients were randomly assigned (1:1) via an interactive response system to receive once daily oraly administered treatment with mavacamten (starting dose 5 mg) or placebo for 30 weeks.
	Allocation concealment (selection bias)	Low Risk	Patients were randomly allocated via an interactive response system with sufficient masking taking place. Drug containers were of identical appearance.
	Blinding of participants and personnel (performance bias)	Low Risk	Participants and Investigators were blinded and the outcome is not likely to be influenced by the blinding.
	Blinding of outcome assessment (detection bias)	Low Risk	Outcome is not likely to be influenced by the blinding.
	Incomplete outcome data (attrition bias)	Low Risk	No missing outcome data observed.
	Selective reporting (reporting bias)	Low Risk	All pre-specified endpoints were reported.
	Other bias	Low Risk	The study appears to be free of other sources of bias.
**VALOR-HCM TRIAL**	Random sequence generation (selection bias)	Unclear Risk	Insufficient information to assess whether an important risk of bias exist.

	Cochrane Risk-of-Bias Tool		
	Bias	Risk of bias	Author judgement
	Allocation concealment (selection bias)	Unclear Risk	Insufficient information to assess whether an important risk of bias exists. Method of concealment has not been described.
	Blinding of participants and personnel (performance bias)	Low Risk	Investigators were blinded and the outcome is not likely to be influenced by the blinding.
	Blinding of outcome assessment (detection bias)	Low Risk	Statistical analyses were performed by an independent statistician.
	Incomplete outcome data (attrition bias)	Low Risk	No missing outcome data observed.
	Selective reporting (reporting bias)	Low Risk	All pre-specified endpoints were reported.
	Other bias	Low Risk	The study appears to be free of other sources of bias.
**MAVERICK TRIAL**	Random sequence generation (selection bias)	Unclear Risk	Insufficient information to assess whether an important risk of bias exist
	Allocation concealment (selection bias)	Unclear Risk	Insufficient information to assess whether an important risk of bias exists. Method of concealment has not been described.

	**Cochrane Risk-of-Bias Tool**		
	Bias	Risk of bias	Author judgement
	Blinding of participants and personnel (performance bias)	Low Risk	Investigators were blinded and the outcome is not likely to be influenced by the blinding.
	Blinding of outcome assessment (detection bias)	Low Risk	Outcome is not likely to be influenced by the blinding.
	Incomplete outcome data (attrition bias)	Low Risk	No missing outcome data observed.
	Selective reporting (reporting bias)	Low Risk	All pre-specified endpoints were reported.
	Other bias	Low Risk	The study appears to be free of other sources of bias.
